# Clinical presentation domains and venous anatomical patterns in cerebral venous thrombosis: a retrospective cohort study

**DOI:** 10.1186/s12883-026-05061-7

**Published:** 2026-06-11

**Authors:** Amirreza Radfar, Etrat Hooshmandi, Vahid Reza Ostovan, Sedighe Hooshmandi, Maryam Poursadeghfard, Nima Fadakar, Mahnaz Bayat

**Affiliations:** 1 Clinical Neurology Research Center, Shiraz University of Medical Sciences, Shiraz, Iran; 2 Medical Imaging Research Center, Shiraz University of Medical Sciences, Shiraz, Iran

**Keywords:** Cerebral venous thrombosis, Venous anatomical involvement, Clinical presentation, Phenotyping, Clinical–anatomical correlation

## Abstract

**Background:**

Cerebral venous thrombosis (CVT) presents with heterogeneous clinical manifestations that may partly reflect differences in venous anatomical involvement. However, systematic evaluation of structured clinical presentation domains in relation to distinct venous involvement patterns remains limited.

**Methods:**

We conducted a retrospective observational cohort study of consecutive adult patients with radiologically confirmed CVT at a tertiary referral center between 2017 and 2025. Presenting symptoms were categorized into predefined, non-mutually exclusive clinical presentation domains, and venous anatomical involvement was classified as isolated superficial thrombosis, isolated parenchymal thrombosis, or combined superficial and parenchymal thrombosis. Domain distributions and overall presentation burden were compared across anatomical patterns, and associations between clinical domains and venous involvement were examined using multinomial logistic regression.

**Results:**

Among 325 patients (median age 39.4 years; 68% female), superficial venous involvement was observed in 68.9%, combined involvement in 22.5%, and isolated parenchymal thrombosis in 8.6%. Intracranial hypertension–related presentation was independently associated with combined thrombosis compared with isolated superficial involvement (adjusted OR 3.30, 95% CI 1.07–10.14). Visual presentation was inversely associated with isolated parenchymal thrombosis (adjusted OR 0.20, 95% CI 0.04–0.94). Other clinical domains, including cortical irritation–related presentation and deep cerebral dysfunction, were not independently associated with venous anatomical patterns. Overall presentation burden did not differ significantly across anatomical groups.

**Conclusion:**

Predefined clinical presentation domains show selective but limited associations with venous anatomical patterns in CVT. Intracranial hypertension–related presentation was associated with combined venous involvement, whereas the inverse association between visual presentation and isolated parenchymal thrombosis should be interpreted cautiously. Substantial clinical overlap across anatomical groups underscores the complex and multifactorial nature of clinical–anatomical relationships in CVT.

**Clinical trial registration:**

Not applicable. This study was an observational retrospective cohort study and was not registered as a clinical trial.

**Supplementary Information:**

The online version contains supplementary material available at 10.1186/s12883-026-05061-7.

## Introduction

Cerebral venous thrombosis (CVT) represents a distinct and uncommon cause of stroke, with an estimated annual incidence of approximately 11–12 cases per million adults [1]. Unlike arterial ischemic stroke, CVT predominantly affects younger individuals, with a median age of approximately 37 years, and demonstrates a marked female predominance [2, 3]. Its clinical presentation is notably heterogeneous, ranging from isolated headache to seizures, focal neurological deficits, and impaired consciousness [4–6]. Although advances in neuroimaging have improved recognition and survival, substantial variability persists in clinical manifestations, and the relationship between presenting symptom patterns and specific anatomical territories of venous involvement remains insufficiently characterized.

The clinical variability observed in CVT likely reflects differences in the anatomical distribution of venous occlusion and the resulting pathophysiological consequences, including venous congestion, parenchymal injury, and intracranial hypertension [7, 8]. Thrombosis involving superficial sinuses, deep venous structures, or combined territories may lead to distinct patterns of brain involvement and symptom expression [9]. While prior studies have described common presenting features and radiologic patterns, structured clinical–anatomical correlations have not been comprehensively evaluated within a unified analytical framework.

Several studies have described specific associations between anatomical involvement and individual clinical manifestations in CVT [10, 11]. Seizures, for example, are more frequently observed in cases involving cortical veins or the superior sagittal sinus and in the presence of hemorrhagic lesions, consistent with cortical surface dysfunction [12]. Conversely, the location of headache does not reliably correspond to the site of sinus occlusion, underscoring the complexity of clinical–anatomical relationships [13]. Similarly, observational cohorts comparing patients with and without cortical vein involvement have reported differences in focal neurological deficits and other presenting features [14]. However, these findings have largely emerged from analyses focused on isolated symptoms or single anatomical sites. A comprehensive evaluation of structured clinical presentation domains across distinct venous involvement patterns has not been systematically undertaken.

In this context, we conducted a retrospective cohort study to evaluate the association between predefined clinical presentation domains and anatomical patterns of venous involvement in patients with CVT. By analyzing symptom clusters within a structured framework and across superficial, deep, and combined thrombosis patterns, we aimed to determine whether consistent clinical–anatomical relationships can be identified beyond isolated symptom associations. Clarifying these relationships may contribute to a more systematic characterization of CVT phenotypes and support more structured diagnostic assessment at presentation.

## Methods

### Study design and setting

This retrospective observational cohort study was conducted at a tertiary referral center and included adult patients diagnosed with cerebral venous thrombosis (CVT). Consecutive cases were identified through systematic review of institutional clinical and radiological databases between 2017 and 2025. The study was designed to evaluate associations between predefined clinical presentation domains and anatomical patterns of venous involvement.

### Study population and eligibility

All adult patients with radiologically confirmed CVT were screened for inclusion. Diagnosis required a compatible clinical presentation together with objective evidence of venous thrombosis on computed tomography venography, magnetic resonance venography, or cerebral angiography. Patients were excluded if clinical documentation was incomplete, if imaging quality or availability was insufficient to permit reliable classification of venous anatomical involvement, or if key demographic or clinical variables required for multivariable analyses were missing. Of 360 patients initially screened, 35 were excluded for these reasons, resulting in 325 patients with complete clinical, imaging, and covariate data included in the final analytic cohort.

### Clinical data collection

Demographic characteristics, presenting symptoms, neurological findings, and imaging features were retrospectively extracted from electronic medical records using a standardized data abstraction framework. Clinical data reflected findings at initial presentation and during the index hospitalization and were reviewed for internal consistency and completeness prior to analysis. Level of consciousness at admission was assessed using the Glasgow Coma Scale (GCS), a previously published and widely used clinical scale for evaluating impaired consciousness, rather than a scale developed specifically for this study [15].

### Clinical presentation domains

Presenting symptoms and neurological signs were grouped a priori into study-defined clinical presentation domains informed by established cerebral venous thrombosis (CVT) syndromic descriptions and contemporary guideline summaries of common presenting manifestations. Established CVT frameworks generally describe syndromes related to intracranial hypertension, focal neurological involvement, diffuse encephalopathy, and cavernous sinus involvement, while recent guideline summaries list common presenting features including headache, seizures, focal neurological deficits, depressed level of consciousness/encephalopathy, visual loss, and diplopia or other cranial neuropathies.

To reduce subjectivity in domain classification, all cases were independently reviewed by two neurologists blinded to imaging findings, with discrepancies resolved through consensus. Although formal inter-rater reliability metrics were not calculated, the use of predefined criteria and independent review by blinded clinicians aimed to minimize classification bias.

Accordingly, we classified presentation into the following non-mutually exclusive domains: intracranial hypertension–related presentation (headache, papilledema, or other features suggestive of raised intracranial pressure in the absence of focal deficits); cortical irritation–related presentation (epileptic seizures and/or focal neurological deficits considered clinically consistent with cortical involvement); deep cerebral dysfunction/altered consciousness (reduced level of consciousness, encephalopathy, or diffuse cognitive impairment); visual presentation (visual disturbance, visual field deficit, or visual loss); cranial nerve–related presentation (documented cranial nerve palsies, including ophthalmoplegia and other cranial neuropathies); and vestibular/posterior fossa–related presentation (vertigo, dizziness, gait instability, imbalance, or other symptoms clinically suggestive of posterior fossa involvement). The visual presentation domain was retained as a composite variable because the registry did not systematically distinguish between visual manifestations potentially related to elevated intracranial pressure, such as blurred vision or diplopia, and focal visual deficits, such as visual field cuts or visual loss. Therefore, separate analyses of visual symptom subtypes were not feasible. These domains were intended to operationalize established CVT symptom patterns into analyzable clinical groupings for this cohort rather than to reproduce a universally accepted formal classification [16–18].

### Presentation burden

To describe overall clinical complexity at presentation, a presentation burden score was calculated for each patient as the total number of clinical presentation domains present, with possible values ranging from 0 to 6. This measure was used to explore whether overall presentation complexity differed across venous anatomical involvement patterns. No imputation was performed, and burden was calculated only in patients with complete domain information.

### Venous anatomical involvement

Venous anatomical involvement was classified on the basis of venographic imaging findings into three mutually exclusive patterns: isolated superficial venous thrombosis, isolated parenchymal venous thrombosis defined as involvement of cortical and/or deep cerebral veins, and combined superficial and parenchymal venous thrombosis [9]. Classification was based primarily on formal radiology reports and was supplemented, when necessary, by direct review of imaging studies to ensure consistency with predefined anatomical criteria.

### Statistical analysis

Continuous variables are presented as medians with interquartile ranges, and categorical variables as counts and percentages. Comparisons of clinical presentation domain frequencies across venous anatomical involvement patterns were performed using chi-square or Fisher’s exact tests, as appropriate. Differences in presentation burden across venous anatomical patterns were assessed using the Kruskal–Wallis test. Associations between individual clinical presentation domains and venous anatomical involvement patterns were evaluated using multinomial logistic regression, with isolated superficial venous thrombosis as the reference category. Multivariable models were adjusted a priori for sex and age greater than 37 years. To assess the robustness of associations to imaging modality, a sensitivity analysis was performed including only patients who underwent MRI and MR venography. The age threshold of 37 years was selected based on the reported median age of CVT in large epidemiological cohorts, allowing stratification around the typical age distribution of the disease. Given the exploratory nature of the study and the limited size of certain anatomical subgroups, covariate adjustment was intentionally restricted to key demographic variables to reduce the risk of model overfitting. Results are reported as odds ratios with 95% confidence intervals. Statistical significance was defined as a two-sided p value < 0.05.

### Statistical software

All statistical analyses were performed using IBM SPSS Statistics (IBM Corp., Armonk, NY, USA). Figures were generated using Python with standard scientific visualization libraries.

## Results

### Study cohort and baseline characteristics

The final analytic cohort comprised 325 patients with radiologically confirmed cerebral venous thrombosis and complete clinical and imaging data. The median age at admission was 39.4 years (IQR 15.1), and 68% of patients were female. Most patients presented with preserved consciousness, with a median Glasgow Coma Scale score of 15 (IQR 13–15). In-hospital mortality occurred in 8% of cases. Magnetic resonance imaging with venography was the predominant diagnostic modality, with MR venography performed in 78.2% of patients.

Superficial venous system involvement was the most frequent anatomical pattern, observed in 68.9% of cases, followed by combined superficial and parenchymal involvement in 22.5% and isolated parenchymal involvement in 8.6%. Baseline demographic, clinical, and imaging characteristics are summarized in Table 1.

**Table 1:** Baseline demographic, clinical severity, imaging, and radiologic characteristics of the study cohort

Characteristic	*N*	%
Age at admission, years, median (IQR)	39.4 (15.1)	—
Female sex	221	68
GCS at admission, median (IQR)	15 (13–15)	—
In-hospital mortality	26	8
Imaging modalities performed		
CT without contrast	265	81.5
CT with contrast	8	2.5
MRI	259	79.7
CT venography (CTV)	71	21.8
MR venography (MRV)	254	78.2
Cerebral angiography	22	6.8
CT imaging findings†		
Venous thrombosis	65	20
Venous infarction	95	29.2
Intracranial hemorrhage	110	33.8
MRI imaging findings†		
Venous thrombosis	172	52.9
Venous infarction	89	27.4
Intracranial hemorrhage	99	30.5
MRI signs of raised intracranial pressure*	3	0.9
Venous anatomical involvement pattern		
Superficial venous system only	224	68.9
Mixed superficial + deep/cortical	73	22.5
Non-superficial only (deep and/or cortical)	28	8.6

†Imaging findings are reported irrespective of imaging modality availability; patients in whom a modality was not performed are included in the denominator unless otherwise specified

*MRI signs of raised intracranial pressure were defined as radiological findings suggestive of increased intracranial pressure, including optic nerve sheath distension, posterior globe flattening, empty or partially empty sella, or transverse sinus stenosis when explicitly reported on MRI/MRV. This imaging variable was distinct from the clinical intracranial hypertension–related presentation domain, which included symptoms or signs such as headache and papilledema

### Clinical presentation and presentation domain distribution

Presenting symptoms were heterogeneous. Headache was the most frequent symptom, reported in 88% of patients, followed by seizures in 34.5% and decreased level of consciousness in 33.8%. Motor deficits were observed in 30.2% of cases, and visual symptoms were reported in approximately one-quarter of patients. Detailed presenting symptoms and neurological findings are provided in Supplementary Table S1.

When categorized into predefined clinical presentation domains, intracranial hypertension–related presentation was the most common domain overall, followed by cortical irritation–related presentation and deep cerebral dysfunction or altered consciousness. The distribution of clinical domains differed across venous anatomical involvement patterns (Table 2).

**Table 2:** Distribution of clinical syndromic domains according to venous anatomical involvement pattern

Clinical syndromic domain	isolated superficial venous thrombosis (*n* = 224) n (%)	Isolated parenchymal venous thrombosis (*n* = 28) n (%)	Combined superficial and parenchymal venous thrombosis (*n* = 73) n (%)	*p* value
Intracranial hypertension–related presentation	196 (87.5)	21 (75.0)	69 (94.5)	0.024
Cortical irritation–related presentation (focal deficit and/or seizures)	117 (52.2)	22 (78.6)	45 (61.6)	0.018
Deep cerebral dysfunction / altered consciousness	71 (31.7)	14 (50.0)	25 (34.2)	0.155
Visual presentation syndrome	64 (28.6)	2 (7.1)	12 (16.4)	0.01
Cranial nerve–related presentation	26 (11.6)	4 (14.3)	4 (5.5)	0.261
Vestibular–brainstem–related presentation	62 (27.7)	8 (28.6)	17 (23.3)	0.744

**Fig. 1:**
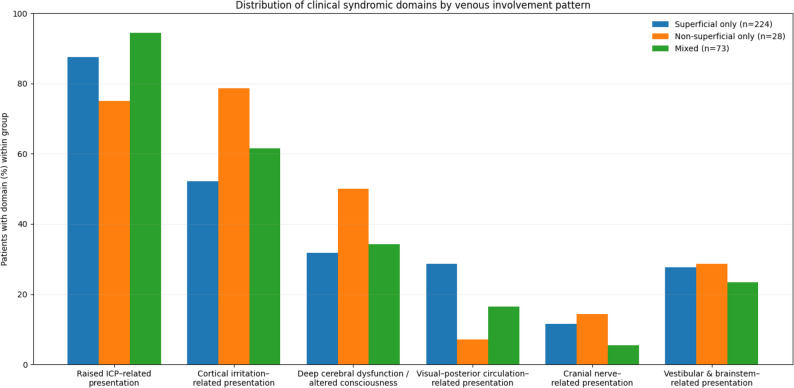
Distribution of clinical syndromic domains across venous anatomical involvement patterns

Intracranial hypertension–related presentation was more frequent among patients with combined superficial and parenchymal venous thrombosis compared with those with isolated superficial involvement (94.5% vs. 87.5%). Cortical irritation–related presentation was most common in patients with isolated parenchymal venous thrombosis (78.6%). In contrast, visual presentation was less frequent among patients with isolated parenchymal venous involvement (7.1%) compared with other groups. No statistically significant differences were observed for cranial nerve–related or vestibular–brainstem–related presentations across anatomical patterns. These distributions are illustrated in Fig. 1.

### Associations between clinical presentation domains and venous anatomical involvement

**Fig. 2:**
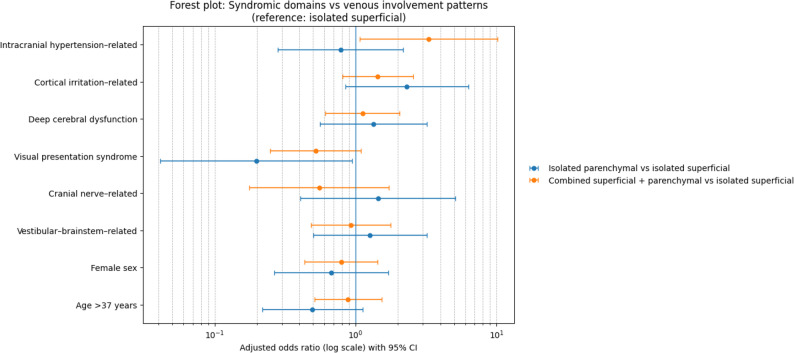
Forest plot of multinomial logistic regression illustrating associations between clinical presentation domains and venous anatomical involvement patterns in cerebral venous thrombosis

In multinomial logistic regression analyses adjusted for sex and age greater than 37 years, selected clinical presentation domains demonstrated differential associations with venous anatomical involvement patterns (Table 3; Fig. 2).

**Table 3:** Multinomial logistic regression of venous anatomical involvement patterns according to clinical presentation domains

Predictor	Isolated parenchymal venous thrombosis vs. isolated superficial venous thrombosis OR (95% CI)	*p* value	Combined superficial and parenchymal venous thrombosis vs. isolated superficial venous thrombosis OR (95% CI)	*p* value
Intracranial hypertension–related presentation	0.78 (0.28–2.18)	0.642	3.30 (1.07–10.14)	0.037
Cortical irritation–related presentation (focal deficit and/or seizures)	2.31 (0.85–6.31)	0.102	1.44 (0.81–2.56)	0.219
Deep cerebral dysfunction / altered consciousness	1.34 (0.56–3.20)	0.51	1.12 (0.61–2.06)	0.711
Visual presentation syndrome	0.20 (0.04–0.94)	0.042	0.52 (0.25–1.09)	0.085
Cranial nerve–related presentation	1.45 (0.41–5.13)	0.569	0.55 (0.18–1.72)	0.306
Vestibular–brainstem–related presentation	1.27 (0.50–3.20)	0.617	0.93 (0.48–1.77)	0.815
Female sex	0.67 (0.26–1.70)	0.401	0.79 (0.44–1.44)	0.439
Age > 37 years	0.50 (0.22–1.12)	0.092	0.89 (0.51–1.54)	0.669

Intracranial hypertension–related presentation was independently associated with increased odds of combined superficial and parenchymal venous thrombosis compared with isolated superficial thrombosis (OR 3.30, 95% CI 1.07–10.14, *p* = 0.037). Visual presentation was inversely associated with isolated parenchymal venous thrombosis compared with superficial involvement (OR 0.20, 95% CI 0.04–0.94, *p* = 0.042).

Other domains, including cortical irritation–related presentation, deep cerebral dysfunction or altered consciousness, cranial nerve–related presentation, and vestibular–brainstem–related presentation, were not independently associated with venous anatomical patterns after adjustment. Neither female sex nor age greater than 37 years demonstrated significant independent associations in the adjusted models. Overall model performance indicated modest explanatory capacity. A sensitivity analysis restricted to patients who underwent both MRI and MR venography (*n* = 220) demonstrated similar frequencies of predefined clinical presentation domains compared with the full cohort. Intracranial hypertension–related presentation was observed in 88.2% versus 88.0% of patients in the full cohort, cortical irritation–related presentation in 54.1% versus 56.6%, deep cerebral dysfunction in 32.1% versus 33.8%, visual presentation in 27.3% versus 24.1%, cranial nerve–related presentation in 10.9% versus 10.4%, and vestibular/posterior fossa–related presentation in 28.2% versus 26.7%, respectively. These findings suggest that the results were not materially influenced by imaging modality.

The clinical presentation burden, defined as the total number of affected clinical domains, was similar across venous anatomical involvement patterns (mean rank: isolated superficial = 162.95, isolated parenchymal = 175.38, combined = 158.42; Kruskal–Wallis H = 0.72, df = 2, *p* = 0.698) and is reported in Supplementary Table S2.

## Discussion

In this retrospective cohort of 325 patients with cerebral venous thrombosis, we systematically evaluated the association between predefined clinical presentation domains and anatomical patterns of venous involvement. Intracranial hypertension–related presentation was associated with combined superficial and parenchymal thrombosis (adjusted OR 3.30, 95% CI 1.07–10.14). Conversely, the composite visual presentation domain was significantly less frequent in isolated parenchymal thrombosis (adjusted OR 0.20, 95% CI 0.04–0.94). Given the heterogeneous nature of this domain, which included both symptoms potentially related to intracranial hypertension and focal visual deficits, this finding should be interpreted cautiously. Other symptom domains, including cortical irritation–related presentation and deep cerebral dysfunction, were not independently associated with venous anatomical patterns after adjustment. Importantly, overall presentation burden did not differ across anatomical groups, indicating that anatomical extent does not necessarily translate into greater clinical complexity. Collectively, these findings suggest that while selected symptom domains may exhibit preferential associations with specific venous territories, the clinical–anatomical relationship in CVT is characterized by substantial overlap and limited discriminatory capacity.

Clinical descriptions of CVT have long emphasized its broad and often overlapping symptom spectrum, in which headache, seizures, focal neurological deficits, and signs of raised intracranial pressure frequently coexist and may evolve over time, complicating attempts to infer anatomical localization from presentation alone [19, 20]. Nevertheless, several cohort studies have demonstrated that specific venous territories may influence symptom expression. Liang et al. reported that patients with concomitant cortical vein thrombosis exhibited significantly higher frequencies of seizures (63.0% vs. 11.8%), focal neurological deficits (44.4% vs. 5.9%), and disorders of consciousness compared with those without cortical vein involvement [21]. Similarly, Kalita and colleagues, in a clinico-radiological study of deep cerebral venous thrombosis, observed that patients with combined deep venous and sinus involvement had a more acute clinical course and higher rates of vomiting and papilledema compared with isolated deep venous thrombosis [22]. These findings support the notion that venous territory contributes meaningfully to clinical phenotype. In our cohort, although cortical irritation–related presentation was common in isolated parenchymal involvement (78.6%), this association did not remain independently significant after multivariable adjustment, and overall presentation burden did not differ across anatomical groups. Together, these observations suggest that while certain anatomical patterns may predispose to particular manifestations, the clinical–anatomical relationship in CVT is influenced by overlapping mechanisms and cannot be explained solely by venous distribution.

The pathophysiological consequences of venous congestion and parenchymal injury in cerebral venous thrombosis extend beyond simple tissue infarction. Cytotoxic and vasogenic edema, secondary ischemic cascades, and disruption of neurovascular coupling can contribute to overlapping clinical manifestations across different anatomical patterns. These processes share common molecular and cellular pathways with other forms of stroke and neural injury, including excitotoxicity, inflammatory responses, and glial activation. Structural parenchymal alterations may influence interhemispheric interactions, network connectivity, and even gene expression through mechanisms such as alternative splicing, which can affect recovery dynamics and clinical complexity [23, 24]. This mechanistic context helps explain why similar clinical syndromes can arise despite heterogeneous anatomical involvement.

The selective and modest associations observed in our analysis likely reflect the multifactorial pathophysiology underlying venous outflow obstruction in CVT. In contrast to arterial ischemic stroke, where discrete arterial territories often produce relatively predictable syndromic patterns, venous thrombosis may lead to a dynamic interplay of venous congestion, elevated intracranial pressure, cytotoxic and vasogenic edema, and secondary hemorrhagic transformation [25, 26]. The clinical expression of a given venous territory is further influenced by collateral drainage capacity, the tempo of thrombus evolution, and the extent of parenchymal involvement [27]. As a result, similar anatomical patterns may manifest with heterogeneous symptom profiles, while distinct venous territories may generate overlapping clinical features [25, 28]. This complexity provides a plausible explanation for our findings, in which intracranial hypertension–related presentation was associated with combined thrombosis patterns, whereas other domains did not retain stable anatomical specificity after multivariable adjustment. However, because headache alone contributed to classification within the intracranial hypertension domain, this association should be interpreted in the context of the domain’s limited specificity.

Structural brain alterations associated with deep cerebral dysfunction in CVT may have implications beyond acute presentation, including long-term cognitive and functional outcomes. Recent large-scale morphometric research demonstrates that subtle changes across distributed cortical and subcortical networks, not just focal lesions, can predict deficits in memory, attention, and executive function long after the index event [29]. Similarly, high-resolution connectomic analyses reveal that disruptions in structural and functional brain networks are linked to domain-specific cognitive impairments and neurobehavioral variability following brain injury [30]. Although our study focused on acute clinical presentation rather than long-term outcomes, these findings highlight the potential value of incorporating detailed neuroimaging metrics and advanced morphometric analyses in future CVT research to better understand the relationship between anatomical patterns, neural network disruption, and complex cognitive profiles.

Several limitations of this study should be acknowledged. First, the retrospective single-center design may introduce selection and information bias, and the clinical presentation domains were derived from existing medical records rather than prospectively standardized assessments. Formal inter-rater reliability for the classification of clinical domains and imaging patterns was not evaluated, limiting external validation of the categorization. Second, although the overall cohort was substantial, certain anatomical subgroups, particularly isolated parenchymal venous thrombosis, were relatively small (*n* = 28), resulting in wide confidence intervals and unstable point estimates; findings in these subgroups should therefore be interpreted as exploratory and hypothesis-generating. Third, the presentation burden score assigned equal weight to all clinical domains and has not been formally validated, which may obscure clinically meaningful differences and attenuate detection of associations across anatomical patterns. Fourth, the intracranial hypertension–related domain may overestimate true ICP-related presentations because some patients were classified based on headache alone without additional clinical signs such as papilledema, diplopia, positional headache, or radiological evidence of raised intracranial pressure. Because headache is common but nonspecific in CVT, this classification approach may have reduced the specificity of the intracranial hypertension–related domain and introduced misclassification bias. Fifth, the visual presentation domain combined heterogeneous manifestations, including symptoms potentially related to elevated intracranial pressure, such as blurred vision or diplopia, and focal visual deficits, such as visual field cuts or visual loss. Because individual visual symptom subtypes were not systematically recorded in the registry, we could not determine whether the observed association with visual presentation was driven primarily by ICP-related mechanisms or by focal visual pathway involvement. Findings related to the visual presentation domain should therefore be interpreted cautiously. Sixth, because of the retrospective nature of the study, detailed etiology and thrombophilia data were not fully available, and the multivariable models were adjusted only for age and sex, leaving residual confounding from unmeasured clinical or radiological factors, including venous hemodynamics and extent of parenchymal vein involvement, unaccounted for. Finally, external validation in an independent cohort was not performed, and generalizability to other populations or healthcare settings remains to be established.

## Conclusion

In this retrospective cohort of patients with cerebral venous thrombosis, predefined clinical presentation domains demonstrated selective but limited associations with anatomical patterns of venous involvement. Intracranial hypertension–related presentation was more frequently observed in patients with combined thrombosis patterns, whereas the composite visual presentation domain was less common in isolated parenchymal involvement. Given the heterogeneous composition of the visual domain and the limited specificity of the intracranial hypertension domain, these findings should be interpreted cautiously and considered hypothesis-generating. However, substantial overlap in symptom expression across anatomical territories was observed, and overall presentation burden did not differ between groups. These findings reinforce that CVT phenotypes are shaped by, but not determined solely by, venous anatomical distribution, underscoring the inherent complexity of clinical–anatomical correlations in this disorder.

## Supplementary Information

Supplementary Material 1.

## Data Availability

The datasets analyzed during the current study are not publicly available due to institutional data protection policies but are available from the corresponding authors upon reasonable request.
