# Supplementary material for: Clinical presentation domains and venous anatomical patterns in cerebral venous thrombosis: a retrospective cohort study
Source: BMC Neurol. 2026 Jun 11;26:520. doi: 10.1186/s12883-026-05061-7 (PMC13480053; doi:10.1186/s12883-026-05061-7)
Supplement: Supplementary file 1 [file 12883_2026_5061_MOESM1_ESM.docx]

| **Supplementary Table S1. Detailed presenting symptoms and neurologic findings** | | | |
| --- | --- | --- | --- |
| **Domain** | **Variable** | **N** | **%** |
| Headache | Headache present | 286 | 88 |
|  | Laterality – Bilateral | 170 | 52.3 |
|  | Laterality – Unilateral | 91 | 28 |
|  | Laterality unspecified | 25 | 7.6 |
| Consciousness | Decreased level of consciousness | 110 | 33.8 |
| Visual symptoms | | 78 | 24 |
| Seizure | Seizure present | 112 | 34.5 |
|  | First clinical presentation with seizure | 88 | 27.1 |
|  | Seizure during hospital course | 45 | 13.8 |
|  | Type – Generalized onset | 87 | 26.8 |
|  | Type – Focal onset | 18 | 5.5 |
|  | Type – Unknown onset | 7 | 2.1 |
| Motor | Motor symptoms present | 98 | 30.2 |
|  | Laterality – Unilateral | 72 | 22.2 |
|  | Laterality – Bilateral | 19 | 5.8 |
|  | Type – Hemiparesis | 66 | 20.3 |
|  | Type – Quadriparesis | 13 | 4 |
|  | Type – Monoparesis | 9 | 2.8 |
|  | Type – Paraparesis | 3 | 0.9 |
| Sensory symptoms | | 31 | 9.5 |
| Aphasia | | 46 | 14.2 |
| Cranial nerve involvment | Any cranial nerve palsy | 34 | 10.5 |
|  | Ophthalmoplegia | 14 | 4.3 |
|  | Trigeminal nerve (CN V) | 2 | 0.6 |
|  | Facial nerve (CN VII) | 18 | 5.5 |
|  | Hearing loss (CN VIII) | 1 | 0.3 |
| Dizziness | | 39 | 12 |
| Vertigo | | 53 | 16.3 |
| Abnormal gait | | 25 | 7.7 |
| Other symptoms | | 90 | 27.7 |

| **Supplementary Table S2. Clinical presentation burden according to venous anatomical involvement pattern** | | |
| --- | --- | --- |
| **Venous anatomical involvement pattern** | **N** | **Mean rank of presentation burden** |
| Isolated superficial venous thrombosis | 224 | 162.95 |
| Isolated parenchymal venous thrombosis (cortical and/or deep) | 28 | 175.38 |
| Combined superficial and parenchymal venous thrombosis | 73 | 158.42 |
| Overall comparison (Kruskal–Wallis test) | — | H = 0.72, df = 2, p = 0.698 |
